# From the Intersection of Food-Borne Zoonoses and EU Green Policies to an In-Embryo One Health Financial Model

**DOI:** 10.3390/foods11182736

**Published:** 2022-09-06

**Authors:** Alessandra Mazzeo, Patrizio Tremonte, Silvia Jane Lombardi, Costantino Caturano, Arianna Correra, Elena Sorrentino

**Affiliations:** Department of Agricultural, Environmental and Food Sciences, University of Molise, Via De Sanctis snc, 86100 Campobasso, Italy

**Keywords:** One Health, food-borne zoonoses, EU Green Deal, Farm to Fork, food safety, antimicrobial resistance, economic health, One Health Financial Model, devastating impact of war

## Abstract

The European Union (EU) adopts the One Health (OH) approach, based on the relationships between human, animal, and environmental health. OH concerns a multitude of aspects, some of which are discussed here. OH overlaps the European Green Deal plan and its relaunched Farm to Fork Strategy, which aims at spreading organic farms adopting the circular economy, in order to improve human health through both better environmental conditions and healthier food. Nevertheless, zoonoses cause sanitary cost in terms of infected farm personnel, lower productivity, and lower fertility of infected farm animals. In such scenarios, the decreased breeding yield and the lower income induce higher cost of farm products, meaning that the market price rises, becoming uncompetitive when compared to the prices of industrial products. Consequently, lower revenues can hinder the farm growth expected in the framework of the EU Green Deal. Since zoonosis control is a key element in aligning EU policies aimed at achieving the EU Green Deal goal of “ZERO environmental impact” by 2050, the authors suggest the inclusion of the parameter economic health in the OH approach, in order to individuate EU Member States (MSs) economically unable to conduct eradication programmes and to finance them. Economic health is here considered as a starting point of the new ethical and science-based One Health Financial Model that the authors suggest as an in-embryo model, in which specific rules should regulate public funds, private investments, and trading, which should exclusively concern public services and private enterprises complying with most of the OH parameters. In this way, economic losses due to collateral negative effects deriving from human activities can be progressively decreased, and the entire planet will benefit from the process. Despite the considerable efforts being carried out in the context of the OH approach, war causes tragic and devastating effects on the physical and mental health of human beings, on their lives, on pandemic and zoonotic threats, on animals, on plants and, last but not least, on the environment. War is incompatible with OH. Enormous efforts for peace are therefore urgently needed.

## 1. Introduction

Zoonotic agents are pathogens with an unrestricted host spectrum. In nature, their survival occurs in reservoir animal species, which generally do not present clinical symptoms and, therefore, are difficult to identify. Promiscuity between farmed animals and wildlife increases the risk of transmission of pathogens and their consequent adaptation to new host species, including human beings. Therefore, promiscuity increases the risk of emergence of new zoonoses. According to the World Organisation for Animal Health (OIE), zoonoses represent 60% of human infectious diseases and 75% of the emerging ones; 80% of pathogens of animal origin have strong potential as bioterrorism agents [1]. Deforestation and destruction of natural areas produce promiscuity, pushing wild species to invade new areas and to arrive in anthropic environments. In high-income countries, domesticated animals are as much a potential reservoir of high-risk zoonoses as the wildlife animals in equatorial rainforests or wet markets.

Companion and zoo animals—with limited syndromic monitoring in place—remain an underestimated but potentially high-risk disease reservoir for emerging zoonoses [2]. Through the global commercialisation of food, food-borne zoonoses (FZs) can also reach individuals who have never been in contact with infected animals or their environment.

FZs are transmitted to human beings indirectly, both through food obtained from infected animals—which are contaminated at their origin—and through food previously contaminated in the various steps of production, sale, and domestic use. Once infected, consumers generally become a source of infection for animals and humans, as well as a source of contamination for food and the environment.

Human beings, animals, and the environment constitute a cohesive and inextricable system, in which human and animal health are interdependent and linked to the health of the ecosystem in which they live. Therefore, they must be considered under the framework of One Health (OH) [1].

Since organic farming contributes to environmental and climate protection, long-term soil fertility, high levels of biodiversity, a safe environment, and high animal welfare standards [3], the European Commission (EC) has set a target of at least 25% of the European Union’s (EU’s) agricultural land being under organic farming by 2030 [4]. To achieve this goal and help organic agriculture reach its maximum potential, the EC proposes an action plan for organic production in the EU [3]. Then, improvement of human health can be achieved through better environmental conditions and healthier food. The EU’s organic logo gives a coherent visual identity to organic products produced in the EU. This makes it easier for consumers to identify EU organic products, and helps farmers to market them [5]. Thus, OH overlaps the European Green Deal plan and its relaunched Farm to Fork Strategy. 

Nevertheless, zoonoses and animal infectious diseases cause decreased breeding yields and reduced income. Consequently, the cost and market price of farm products become uncompetitive with respect to the price of industrial food. In other words, zoonoses cause lower revenues, hindering the growth of organic farming expected in the framework of the EU Green Deal. In such scenarios, zoonosis control becomes a key element to align EU policies aimed at achieving the goal of “ZERO environmental impact” by 2050. 

## 2. One Health

The OH approach is adopted in world policies by:

- The World Health Organization (WHO);
- The Food and Agriculture Organization of the United Nations (FAO) [6];
- The World Organisation for Animal Health (OIE) [1];
- The European Union (EU) [7];
- The USA, where the Centers for Disease Control and Prevention (CDC—Atlanta, GA) host the National Center for Emerging and Zoonotic Infectious Diseases, which works “to protect people at home and around the world from emerging and zoonotic infections ranging from A to Z—anthrax to Zika—since we are living in an interconnected world where an outbreak of infectious disease is just a plane ride away” [8].

Recently, the Global Health Summit, held in Rome in May 2021, stated the need to adopt the OH approach in the *Rome Declaration*, issued at the conclusion of the summit [9]. On 12/12/2021, the FAO, OIE, WHO, and the United Nations Environment Programme (UNEP) adopted the following definition: “One Health is an integrated, unifying approach that aims to sustainably balance and optimize the health of people, animals and ecosystems. It recognizes the health of humans, domestic and wild animals, plants, and the wider environment (including ecosystems) are closely linked and interdependent. The approach mobilizes multiple sectors, disciplines and communities at varying levels of society to work together to foster well-being and tackle threats to health and ecosystems, while addressing the collective need for clean water, energy and air, safe and nutritious food, taking action on climate change, and contributing to sustainable development”.

Although health, food, water, the environment, and energy are very broad topics with specific and sectoral concerns, interdisciplinary and cross-sector collaborations could provide more effective tools both to protect us from emerging infectious diseases and antimicrobial resistance, and to promote the biodiversity and integrity of ecosystems.

Furthermore, OH can help to address the full spectrum of disease control—from prevention to detection (a lesson learned during the ongoing COVID-19 pandemic), through preparedness, response, and management—and to improve and promote health.

The OH approach can be applied at the national, community, regional, and global levels, and relies on shared and effective governance, communication, collaboration, and coordination. Using this approach will make it easier for people to better understand the co-benefits, risks, trade-offs, and opportunities to promote equitable and holistic solutions [10].

Firstly, the One Health High-Level Expert Panel (OHHLEP) advisory board focuses on preparing a policy-relevant scientific assessment of emerging health crises arising from the human–animal–ecosystem interface, on developing a long-term strategic approach to reduce the risk of zoonotic pandemics—with an associated monitoring and early warning framework—and on the synergies necessary to improve and institutionalise the OH approach, as well as in the areas that drive the risk of pandemics [11].

The WHO, OIE, and FAO work together to control and prevent health risks arising from the human–animal–ecosystem interface. They are developing global strategies and tools to ensure a coherent and harmonised approach worldwide, and to better coordinate national and international human, animal, and environmental health policies. In October 2017, the second Tripartite Strategy document was published, with a particular focus on the following aspects: strengthening national services for human and animal health and food safety; strengthening and modernisation of early warning and surveillance/monitoring systems; prediction, preparedness, and response to emerging, re-emerging, and neglected infectious diseases; encouraging and promoting coordinated research to achieve a common understanding of the highest-priority zoonotic diseases; and challenges posed by food safety that require a multisectoral approach in the context of improving food security [12].

The One Health Surveillance Codex (OHS Codex) was established to provide a framework for the OH community to constantly share practical solutions applicable for stakeholders from different One Health Surveillance sectors. The OHS Codex framework includes four high-level action principles, which support, respectively, collaboration, knowledge exchange, data interoperability, and dissemination, which are summarized as follows. (i) The *collaboration* principle represents the need for positive interaction and communication between actors, and it is considered to be the foundation of OH surveillance; without the willingness and ability to collaborate and communicate, surveillance systems will remain sectorial and fragmented. This principle collects tools and resources that facilitate the understanding of surveillance across sectors and disciplines, whilst also showing that these solutions can be found in environments where collaboration works. (ii) The *knowledge* principle represents the need for mutual scientific understanding and expertise in the evolution of knowledge of other sectors; the aim of this principle is to provide surveillance professionals and stakeholders with guidance on sources of knowledge. (iii) The *data* principle represents the ability to understand, reuse, and interpret surveillance data across sectors and disciplines. (iv) The *dissemination* principle represents the distribution of surveillance results to stakeholders and surveillance actors, including industries and policymakers [13].

The EC’s Green Deal is an integral part of the OH strategy to implement the United Nations’ Agenda 2030 and the Sustainable Development Goals (SDGs), which are an urgent and universal call for action by all high- and low-income countries. The EU will work with all partner countries to increase climate and environmental resilience to prevent these challenges from becoming sources of conflict, food insecurity, and forced migration. Some parts of the EU Green Deal, such as the Farm to Fork Strategy and the Biodiversity Strategy, explicitly refer to OH. In recent decades, the OH concept has expanded from the medical and veterinary sciences to include a rapidly growing range of synergistic disciplines, including food safety, food security, public health, health economics, environmental ecosystem health, social sciences, and animal health and welfare. 

It is now recognised that environmental factors—including chemical contaminants in animals and animal products, veterinary drug residues, and pesticides—play significant roles that call for holistic transdisciplinary approaches to move towards safe and sustainable food systems [14].

## 3. Food-Borne Zoonoses and EU Animal Health Laws

Zoonosis control in the EU is regulated by Directive 2003/99/EC [15], which lists in LIST A the zoonoses to be subjected to mandatory control, including the main FZs, such as brucellosis and tuberculosis in cattle and buffaloes, salmonellosis in poultry and turkeys, and trichinellosis. The individual Member States (MSs) activate National Control Plans (NCPs) in primary production. NCPs are mandatory and possibly co-financed by the European Commission [16]. They are harmonised in order to make the results comparable, thanks to methods of analysis developed, validated, and disseminated by the European Union Reference Laboratories (EURLs) [17], which transfer them to individual National Reference Laboratories (NRLs) of each Member State (MS) which, in turn, disseminate them extensively to the laboratories of their own national territory. Other optional plans can be activated, based on the epidemiological situation of specific territories as regards the zoonoses included in LIST B of Directive 2003/99/EC.

The NCPs are based on the following aspects: diagnosis (serological diagnosis is adopted if there is no possibility of taking samples useful for direct diagnosis); identification and elimination of infected animals or of the entire herd hosting them; attribution of the sanitary qualification “Officially Free” (OF) to breeding and, progressively, to the entire province or region and to the MS in which the specific zoonosis has been eradicated; the prohibition of vaccination (generally mandatory); biosecurity measures, which must be adopted in a strict way, because the presence of the NCP causes the absence of natural or artificial immunological responses against the specific zoonotic agent, due to the pathogen-free territory and to the prohibition of vaccination, respectively. While vaccination can be authorised in the event of a serious emergency with attribution of the sanitary qualification “Free” (F), antibiotic therapy is not allowed. In case of vaccine authorisation, the OF qualification can be newly acquired when only non-vaccinated animals are present in livestock. NCPs are divided into “eradication plans”, if the zoonosis is present in the animal population, and “surveillance plans”, which are limited to checking whether the eradicated zoonosis is re-emerging. The surveillance, in turn, is divided into active surveillance, which involves official controls on farms aimed at identifying infected animals, and passive surveillance, which induces the activation of official controls only when suspected cases are signalled.

Data relating to the results obtained in primary production under the application of NCPs, reported human cases, test results in food control activities, test results in feed control activities, and antimicrobial resistance converge in *The European Union One Health Zoonoses Report*, published jointly by the European Food Safety Authority (EFSA) and the European Centre for Disease Prevention and Control (ECDC), in open access and on an annual basis. In the report published in 2021 [18], relating to data for the year 2020, *Salmonella* (Le Minor and Popoff, 1987) [19] remains the most frequently reported etiological agent in episodes of FZs in the EU, and the pathogens considered in relation to the foods of greatest risk were found to be *Salmonella* in eggs and their derivatives, norovirus in crustaceans and molluscs (including bivalves), and *Listeria monocytogenes* (Murray, Webb, and Swan, 1926) [19] in fish and fish products. Correlation between human brucellosis and non-OF territories persists [18].

Regarding *Salmonella* infections, on 17 February 2022, the United Kingdom (UK) reported a cluster of cases with monophasic *Salmonella* Typhimurium sequence type 34 infection. As of 18 May 2022, 324 cases had been reported in 12 EU/EEA countries and the UK, including two distinct strains. As of 3 June 2022, 392 cases of monophasic *S*. Typhimurium have been identified in the EU/EEA and the UK (*n* = 370 confirmed cases and *n* = 22 probable cases). In addition, cases have been identified in Canada (*n* = 4), Switzerland (*n* = 48), and the United States (*n* = 1), bringing the total number of cases to 445 globally [20,21]. Most cases were detected in persons below 10 years of age, and 41% of all cases were hospitalised. The two strains are multidrug-resistant, and some tested isolates also exhibit resistance to disinfectants based on quaternary ammonium compounds and hydrogen peroxide, but are susceptible to azithromycin, ciprofloxacin, meropenem, and third-generation cephalosporins. Epidemiological investigations have suggested some specific chocolate products, produced in a plant in Belgium, as likely vehicles of infection. Two monophasic *Salmonella* Typhimurium strains matching the outbreak strains were identified in the buttermilk line at the Belgian plant between December 2021 and January 2022. The buttermilk was provided by an Italian supplier, where *S.* Typhimurium was not detected. On 8 April 2022, on the basis of official controls, the Belgian food safety authority decided to revoke the production authorisation of the indicated plant due to lack of transparency and insufficient guarantees for safe production. All at-risk products produced at the closed plant have been recalled. National competent authorities in several countries issued public warnings. This outbreak has evolved rapidly, with children most at risk of severe infection. The plant closure and the global recall of all potentially hazardous products have reduced the risk of exposure. However, eight cases cannot be explained by consumption of chocolate products, suggesting that there may also be other sources of infection.

The ECDC has published, in open access, the data on antimicrobial resistance [22] and, jointly with EFSA, *The European Union Summary Report on Antimicrobial Resistance in Zoonotic and Indicator Bacteria from Humans, Animals and Food in 2018/2019* [23].

### 3.1. EU Control Programmes

EU co-funded veterinary programmes have proven to be a catalyst for achieving improvements in public and animal health, reductions in disease prevalence/incidence, safeguarding of public health (in the case of zoonoses), disease prevention/management in the context of the EU Animal Health Strategy, and economic benefits for the EU as whole by protecting the value of the sector, contributing to market stability, guaranteeing safe trade, increasing extra-EU trade, and reducing human health costs.

Significant differences in MSs’ veterinary systems and livestock facilities lead to variability in the implementation of programmes, risking jeopardising the results achieved at the EU level—particularly when dealing with transboundary diseases [24].

Regulation (EU) No 652/2014 laying down provisions for the management of expenditure relating to the food chain, animal health, and animal welfare concerns diseases with impacts on human health, diseases with impacts on animal health (taking into consideration their potential spread and the morbidity and mortality rates in animal populations), diseases and zoonoses that risk being introduced and/or re-introduced into the EU territory from third-party countries, diseases with the potential to generate a crisis situation with serious economic consequences, and diseases with impacts on trade with third-party countries and on intra-EU trade. It also concerns the main FZs, including bovine tuberculosis, bovine brucellosis, echinococcosis, campylobacteriosis, listeriosis, salmonellosis, trichinellosis, and verotoxigenic *Escherichia coli* (Castellani and Chalmers, 1919) infections [19,25].

Regulation (EU) 2016/429 on transmissible animal diseases and amending and repealing certain acts in the area of animal health (“Animal Health Law”) [26] concerns the main FZs, including infection with *Brucella* (Meyer and Shaw, 1920)—specifically, *B. abortus*, *B. melitensis*, and *B. suis*; infection with *Mycobacterium bovis* (Karlson and Lessel, 1970), *M. caprae*, and *M. tuberculosis*, included in the *Mycobacterium tuberculosis* complex; and infestation with *Echinococcus multilocularis* (Leuckart, 1863) [19,27]. It was amended and corrected in 2017, 2018, and 2020 [28]. 

In 2020, in Northern Europe, 20 MSs were officially brucellosis-free in cattle, and 17 MSs were officially tuberculosis-free in cattle, while these zoonoses persisted in the Mediterranean area. Italy, Portugal, and Spain activated co-funded eradication programmes for bovine brucellosis as well as for bovine tuberculosis (also activated in Ireland and Malta) [26], while in Greece only the eradication programme concerning ovine and caprine brucellosis (*B. melitensis*) was co-funded [20,29]. Greece reported the highest prevalence of *Brucella*-positive ruminant herds, and Spain reported the highest prevalence of tuberculosis in cattle.

### 3.2. Control of Non-Regulated Diseases in Cattle and Buffalo (Cattle Diseases Listed under Category C, D, or E in the EU Animal Health Law)

NCPs are in force both for the most important zoonoses and for the most important animal infectious diseases that lack zoonotic potential.

For diseases not included in the European Union Animal Health Law Categories A or B under Commission Implementing Regulation (EU) 2020/2002 [28], approximately one-third of control plans (CPs) are voluntary and can be limited to a well-defined territory of the MS; their funding structure is divided between government and private resources [29].

Countries that have already eradicated diseases such as enzootic bovine leukosis, bluetongue, infectious bovine rhinotracheitis, and bovine viral diarrhoea have also implemented CPs for other diseases in order to further improve the health status of cattle in their country [30], increasing the commercial value of animals and animal products. Consequently, the gap in the health status of farmed animals could progressively increase among the EU MSs.

## 4. The EU Green Policies concerning the Food System

**Figure 1:**
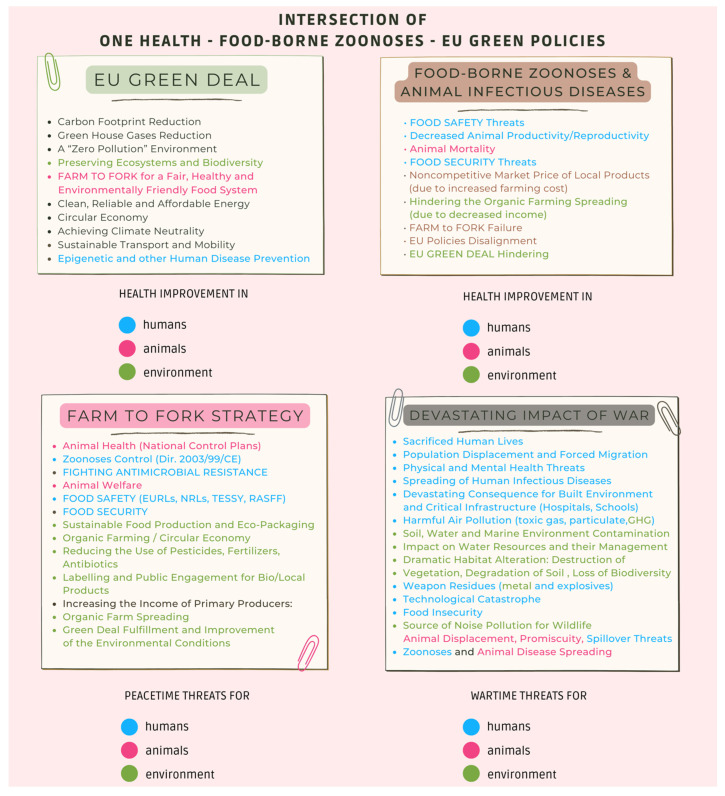
One Health improvements deriving from EU green policies in comparison with peacetime and wartime threats.

In the European Union, OH overlaps the European Green Deal plan launched by the European Commission (EC Green Deal plan) to achieve the goal of “ZERO environmental impact” by 2050. In this scenario, the devastating impact of war must be considered (Figure 1). The Green Deal relaunches the Farm to Fork Strategy for a healthy and environmentally sustainable food system, providing specific measures to make the economy circular, while concomitantly reducing the use of pesticides, fertilisers, and antibiotics, so as to limit the alarming antimicrobial resistance that is spreading worldwide [3,4,31].

### 4.1. Public Engagement

Public engagement can be stimulated through the improvement of food labelling, which should include information concerning the production environment, aimed at facilitating consumers’ choices in the direction of healthy and, at the same time, sustainable diets. A reward mechanism is triggered for farms that adopt the circular economy and that produce in compliance with the objectives of the EU Green Deal. On the other hand, consumers receive beneficial effects on their health, in terms of both food safety and the improvement of environmental conditions [32].

Labelling is a key factor for food safety in agrifood chains. It is often characterised by asymmetric information. Producers and marketers tend to be better informed than consumers about the potential risks of food. The use of innovative strategies to communicate information on food risks can help reduce the divergence between assessed and perceived risks. In this respect, innovative labels—such as traffic-light labels or the use of nanotechnologies—could be valid alternatives. Furthermore, technologies such as Agri-Food 4.0, Blockchain, and the Internet of Things can be useful tools to inform consumers in real time, while also supporting the supply chain decision-making process and improving the coordination process involving farmers, industries, and consumers [33].

### 4.2. Fighting the Antimicrobial Resistance

The Green Deal includes—among the objectives of primary importance—the reduction in the use of antibiotics in livestock production, in order to combat antimicrobial resistance (AMR), which is a global emergency that has increased during the COVID-19 pandemic. This is referred to as the silent, second pandemic [34].

In addition to the wide use of antimicrobials in animals [35], plant agriculture frequently uses antibiotics to enhance crop yields. This means that fruits and vegetables have also become potential sources of AMR [36].

Multidrug resistance (MDR) is continuously expanding worldwide, and poses a challenge in treating infections, necessitating the use of reserve antibiotics, which can have higher cost-to-benefit ratios and a lower safety profile. Among the MDR germs, “the ESKAPE pathogens” have had the greatest impact on healthcare-associated infections—a group of six pathogens with the capacity to elude the bactericidal activity of antibiotics: *Enterococcus faecium* (Orla-Jensen, 1919), *Staphylococcus aureus* (Rosenbach, 1884), *Klebsiella pneumoniae* (Trevisan, 1887), *Acinetobacter baumannii* (Bouvet and Grimont, 1986), *Pseudomonas aeruginosa* (Migula, 1900), and *Escherichia coli* [19]. The ESKAPE group is characterised by pathogenic and transmission, resistance traits—which are represented by enzymatic inactivation, target changes, and alteration of cell permeability through loss of porins or increased expression of efflux pumps—and mechanical protection through biofilm formation [37].

In 2019, an estimated 1.27 million deaths were attributable to bacterial AMR. At the regional level, the all-age death rate attributable to resistance was highest in western sub-Saharan Africa, and lowest in Australasia.

Lower respiratory tract infections accounted for over 1.5 million resistance-associated deaths in 2019, making this the most burdensome infectious syndrome.

The six main pathogens for resistance-associated deaths—*E. coli*, followed by *S. aureus*, *K. pneumoniae*, *Streptococcus pneumoniae* (Chester, 1901), *A. baumannii*, and *Pseudomonas aeruginosa*—were responsible for more than 900,000 deaths attributable to AMR in 2019 [19].

One pathogen–drug combination—methicillin-resistant *S. aureus*—caused more than 100,000 deaths attributable to AMR in 2019, while six others each caused between 50,000 and 100,000 deaths: multidrug-resistant (excluding extensively drug-resistant) tuberculosis, *E. coli* resistant to third-generation cephalosporins and to fluoroquinolones, *A. baumannii* and *K. pneumoniae* resistant to carbapenems, and *K. pneumoniae* resistant to third-generation cephalosporins [38].

In the EU food system, the AMR monitoring and reporting cover the following food-producing animal populations and foods: broilers; laying hens; fattening turkeys; cattle less than one year old; fattening pigs; fresh meat from broilers; and fresh meat from turkeys, pigs, and cattle. 

AMR surveillance concerns the monitoring and reporting of antimicrobial resistance of the following bacteria: *Salmonella* spp., *Campylobacter coli*, *C. jejuni* (Doyle, 1948; Véron and Chatelain, 1973), indicator commensal *E. coli*, *Salmonella* spp., and *E. coli* producing extended-spectrum β-lactamases, AmpC β-lactamases, and carbapenemases. In addition, it may cover indicator commensal *E. faecalis* and *E. faecium* [19,39]. 

In the United States of America, Congress issued the *Disarm Act of 2021* [40], in order to develop an innovative strategy to fight the increase in antimicrobial resistance [41].

## 5. Discussion

NCPs and CPs for animal diseases provide benefits for animals, farmers, producers, and consumers, because they improve animal health and welfare, reduce the use of antibiotics, and—in the case of zoonotic diseases—improve the safety of animal products. NCPs and CPs reduce direct and indirect losses due to diseases. Their implementation involves associated costs for testing and administrative work; however, the cost is usually considered to be outweighed by the benefits [30].

Nevertheless, in non-OF territories, zoonoses and animal infectious diseases cause lower productivity and often lower reproductive capacity of infected farm animals. The decreased breeding yield produces increased cost of farm products that, in turn, induce higher and uncompetitive market prices, e.g., in the framework of Regulation (EC) 853/2004, raw milk must come from cows or buffaloes belonging to a herd that is OF or F from brucellosis. 

In non-OF nor F herds, raw milk may still be used when coming from cows or buffaloes that do not show a positive reaction to tests for brucellosis, nor have any symptoms of the disease. In this situation it is mandatory to have the authorisation of the competent authority in order to make the milk undergo a compulsory heat treatment useful to reveal a negative reaction to the phosphatase test. The cost of additional analyses are generally charged to farmers and, consequently, most of them prefer to discard this milk. Furthermore, infected livestock or infected heads are culled in the frame of zoonosis and animal infectious disease NCPs, and the cost of restocking is not fully covered by indemnities. Thus, zoonoses and animal infectious diseases cause decreased revenues, hindering the growth of organic farms, and hampering the fulfilment of the EU Green Deal objectives [42].

In 2020, EU data on zoonoses and related zoonotic agents reported a drastic decrease in the numbers of human cases [18]. This was evidently influenced not only by the UK’s exit from the EU, but also by the COVID-19 pandemic and the resulting restriction measures imposed in EU MSs. This worldwide event, which today sees the rise of variants of concern (VOCs) of SARS-CoV-2 in selected countries with unconsolidated economies and in which the administration of vaccines is slow, highlights the importance of the OH approach, which is useful in designing a new health system to be applied homogeneously at a global scale—not only as an ethical necessity, but also as an indispensable safeguard to prevent that those who have been left behind from becoming victims and, at the same time, sources of new emergencies [43].

The Economic Community of West African States (ECOWAS) has borne a significant burden of zoonotic disease impacts. To address zoonotic disease threats in ECOWAS, a One Health Zoonotic Disease Prioritization (OHZDP) was conducted in December 2018 to prioritise the zoonotic diseases of greatest regional concern and develop the next steps to address these priority zoonoses through a regional, multisectoral, OH approach.

ECOWAS was the first region to use the OHZDP process to prioritise zoonotic diseases of greatest concern. With the identification of priority zoonotic diseases for the region, ECOWAS member states can collaborate more effectively to address zoonotic disease threats across the region using a OH approach. Strengthening national- and regional-level multisectoral OH coordination mechanisms allow ECOWAS member states to advance OH, and has a significant impact on improving health outcomes for both people and animals living in a shared environment [44].

Since it is not possible to stem the spread of pathogens by constructing disjointed barriers, it becomes imperative to act simultaneously at all levels and on a global scale, financing actions that guarantee uniform conditions of protection. This is necessary to counteract the enormous biological plasticity of microorganisms which, together with their rapid reproduction cycles, makes them easily globalised. In the EU, to limit the spread of zoonoses reported as official cases, actions based on the essential protection of the environment are necessary through the concrete implementation of the Farm to Fork Strategy and the pursuit of the Green Deal; in fact, important zoonoses included in Directive 2003/99 EC, although originally linked to animal reservoirs, can be found today in vegetable foods to be consumed raw, as preferential and hazardous vehicles of transmission to humans [45].

Furthermore, animal diseases threaten global food security. African swine fever (ASF)—a viral haemorrhagic disease characterised by high morbidity and high mortality in domestic and wild swine (but lacking zoonotic potential)—is progressively expanding in Asia and Europe [46]. In particular, in 2017, ASF cases increased in wild boar in Ukraine, [47]; in 2021, ASF continued to be reported in wild boar across Europe, and frequent outbreaks in domestic pigs continued to be reported in Romania, with small numbers of outbreaks also reported in Ukraine, which is currently under military attack [48]. The probable escape of infected wild boars from territories exposed to military attacks or human factors due to the consequences of war (e.g., population displacement) could cause the further spread of outbreaks in neighbouring EU MSs.

This is only a minor consequence of the tragedy of the ongoing war, which sees human lives sacrificed and the mass exodus of the exhausted Ukrainian human population, in whom 32,000 new tuberculosis (TB) cases have been estimated in 2020 (with almost 11,000 cases estimated to be drug-resistant TB), and patients have had to stop their required lengthy treatment [49].

ASF has been present on the island of Sardinia since 1978, without consequences for the Italian mainland, where ASF was later on confirmed on 6 January 2022 in wild boars; the ongoing outbreaks first spread in the regions of Piedmont and Liguria (north-west Italy), and then reached the region of Lazio (Central Italy). Control measures at the event level, including domestic control measures in one domestic outbreak that occurred in a farm hosting nine swine (i.e., disinfection, *ante* and *post mortem* inspections; official disposal of carcasses, byproducts, and waste; zoning; traceability; surveillance within the restricted zone; surveillance outside the restricted zone; stamping out; screening; official destruction of animal products; movement control) and wild control measures (i.e., screening; official disposal of carcasses, byproducts, and waste; movement control; surveillance outside the restricted zone; ante and *post mortem* inspections; official destruction of animal products; surveillance within the restricted zone; zoning) were put in place to avoid the major zooeconomic problems caused by ASF—in particular in the export of fine Italian delicatessen products [50]. The *African swine fever virus* (ASFV), in the genus *Asfivirus* [51], does not induce neutralising antibodies, making difficult the efforts for the production of vaccines. A new candidate vaccine for ASF has been developed recently. It uses an attenuated whole virus which, by inducing antibodies towards many viral antigens, is able to protect swine against infection, but may present the risk of retromutation to the virulent virus. Therefore, the “reversion to virulence” test is an important milestone as part of a series of safety studies. Recently, the U.S. Department of Agriculture’s Agricultural Research Service (ARS) announced that a vaccine candidate for ASF had passed an important safety test required for regulatory approval, moving the vaccine one step closer to commercial availability [52].

Furthermore, Ukraine remains the only country in Europe where rabies is widespread, with about 1600 rabies cases in animals and sporadic cases in humans [53]. Rabies is the deadliest of all known zoonoses and it is lethal to mammals. It negatively impacts on food security and livelihood [54]. Veterinary bodies in countries bordering Ukraine and in other MSs have made exceptions to peacetime restrictions for bringing pets across borders in a bid to aid refugees. Consequently, the risk of introducing rabies in free countries is feared [55].

In Asian countries (e.g., Cambodia, Indonesia, Vietnam), where dogs and cats are used for human consumption, rabies can be considered a food-related zoonosis in workers at dog and cat slaughterhouses and it can be considered a food-borne zoonosis for the consumers of infected dogs and cats. In Vietnam, workers at dog slaughterhouses are vaccinated in the framework of a national programme for rabies control and prevention [56].

There is a strong policy impetus for the OH cross-sectoral approach to address the complex challenge of zoonotic diseases—particularly in low/lower- and middle-income countries (LMICs), where there is limited policy visibility on zoonotic diseases—especially high-burden endemic diseases that disproportionately affect marginalised rural populations [57].

Since 1 January and as of 22 June 2022, 3413 laboratory-confirmed cases and 1 death from monkeypox have been reported to the WHO from 50 countries/territories in 5 WHO regions. In a week, 1310 new cases were reported, and eight new countries reported cases. As of 21 July 2022, 15,848 confirmed cases had been reported. The unexpected appearance of monkeypox and the wide geographic spread of cases indicate that the monkeypox virus might have been circulating below levels detectable by the surveillance systems, and that sustained human-to-human transmission might have been undetected for a period of time [58,59]. In 2020, a total of 4594 suspected cases of monkeypox, including 171 deaths (case fatality rate = 3.7%), have been reported in 127 health zones from 17 out of 26 provinces in the Democratic Republic of the Congo. Communicating monkeypox-related risks and engaging at-risk and affected communities, community leaders, civil society organisations, and healthcare providers—including those at sexual health clinics—in prevention, detection, and care, is essential for preventing further secondary cases and ensuring effective management of the current outbreak [60]. The WHO has been considering declaring monkeypox a Public Health Emergency of International Concern (PHEIC). In 2019, the U.S. Food and Drug Administration (FDA) approved a smallpox and monkeypox vaccine. This is a good example of preparedness [61].

The recent “One Health of Peripheries” proposal highlights violence as a cause of morbidity and mortality, and among the approaches to address its complexity is the prevention of violence against animals. Geographical peripheries are heterogeneous, encompassing entire countries, areas circumscribed within countries, cross-border regions, rural areas, indigenous territories, and favelas. In particular, the contextual effects of favelas on health are mediated by imposed risks and the lack of resources (e.g., money, time, infrastructure, knowledge), creating a vicious circle of vulnerability due to the increased burden of diseases that compromise individuals’ opportunities for economic and social inclusion. The contextual effects of favelas affect multispecies collectives, and this aspect is even more neglected. Animals are exposed and vulnerable to pollution, humidity, darkness, inadequate ventilation, malnutrition, and high population density. It is necessary to promote the health of animals for their own sake, but also for the sake of the humans living with them [62]. Collaboration must be tailored to the surveillance objective and context, characterised by a wide range of factors (i.e., epidemiological, ecological, economic, social, and environmental); successful cross-sectoral collaboration is largely rooted in mutual trust and respect between the different actors [57].

The need for a transition from traditional public health/biosurveillance to a health security intelligence approach to epi/pandemics requires delineating emerging threats from companion, livestock, and wildlife animal communities, which requires funds, the integration of early warning tools, open-source platforms, multisource and multispecies surveillance, proactive diagnostics, field testing technologies, and increased focus on necropsy in captive and wild animals [2]. Diagnostic tools—able to separate immunoglobulin isotypes in order to specifically detect and quantify them simultaneously—could be useful in serological diagnosis aimed at individuating emerging threats, their epidemiological features, and their evolution during vaccination campaigns [63].

Furthermore, OH education must be incorporated into scientific, engineering, and humanities curricula, in order to build capacity in OH skills, with the goal of creating networks that will work to improve public health, food safety, food security, and sustainable agriculture. This can be achieved by establishing new perspectives on the interactions between plants, animals, and humans, recognising the threat of disasters and transboundary diseases to food security. The implementation of OH calls for identifying priority areas for added value of joint activities, and for the effective knowledge elicitation of experts from different and relevant disciplines. Consequently, OH may call for updated models for establishing and maintaining effective and timely collaboration and communication across and within disciplines. The establishment of OH approaches and networks can be of high value for countries aiming to establish or improve their OH activities, supporting science-based regulations in the areas of health, food, and the environment [64].

## 6. Conclusions

Specific evaluation attributes need to be developed to allow the measurement of the impacts and benefits of collaborative surveillance versus a juxtaposition of isolated sectoral surveillance components [65].

In the EU, surveillance systems integrate elements of public and animal health systems and food chains to detect, assess, and control multistate food-borne infections. These systems are based on an investigation and notification approach (Early Warning and Response System-EWRS), the European surveillance portal for infectious diseases EpiPulse, and the Rapid Alert System for Food and Feed (RASFF). The exchange of information is facilitated and coordinated by the EFSA and ECDC, and is followed up by the EC [66].

General requirements for the laboratory and bioinformatic components of whole-genome sequencing (WGS) and associated metadata for food-borne bacteria have been recently published by the International Organization for Standardization. These guidelines provide the basis for harmonisation of bioinformatic analysis and validation of the end-to-end WGS workflows [66,67].

Despite food-borne zoonosis control needs large investments, it can positively affect economies, so the parameter economic health should be included in the OH approach in order to fund eradication programmes in EU MSs facing financial difficulties (heightened by the ongoing COVID-19 pandemic) and, globally, in extra-EU low-income countries, as well as to monitor the effectiveness of interventions and their benefit/cost ratio [42]. EU actions should include, in the case of economic needs, the financing of NCPs in MSs such as Greece which—despite the persistence of the highest notification rate of human cases for brucellosis in the EU, resulting in the highest number of confirmed domestically acquired cases in an MS, and the highest prevalence of *Brucella*-positive ruminant herds - does not benefit from the EC co-funded programme for brucellosis in cattle [18,42].

## 7. Future Directions

In 2012, for the first time, the World Bank highlighted the cumulative societal cost of infectious disease outbreaks. The moment at which a system is able to detect signals of possible threats was correlated with the possible cost of the system. Thus, the generation of high-quality zoonotic situational awareness at the earliest possible stages of an outbreak must be a priority for future health security systems [68]. Promoting the OH approach for food safety (including control of related zoonotic agents) and food security (including control of related animal and plant infectious diseases with no zoonotic potential) is a way to involve the younger generation in entering employment and training in agriculture and the food system. To this end, the obstacle represented by language barriers must be removed. Establishing a broader concept of OH that incorporates the food system as well as cultural and societal awareness is essential. Developing a holistic view of biomedical, biotechnological, and agricultural sciences can help in transforming traditional academics and researchers into OH practitioners who work in transdisciplinary teams to solve complex problems at the interface of human, animal, plant, and environmental health [64].

Given the unequal health status of EU farms with respect to both FZs and infectious animal diseases, it would be appropriate to introduce a new OH assessment parameter: economic health. This could be used to allocate European funds that would allow MSs facing economic difficulties to achieve eradication by reaching the same levels as the MSs with the most advanced animal husbandry.

**Figure 2:**
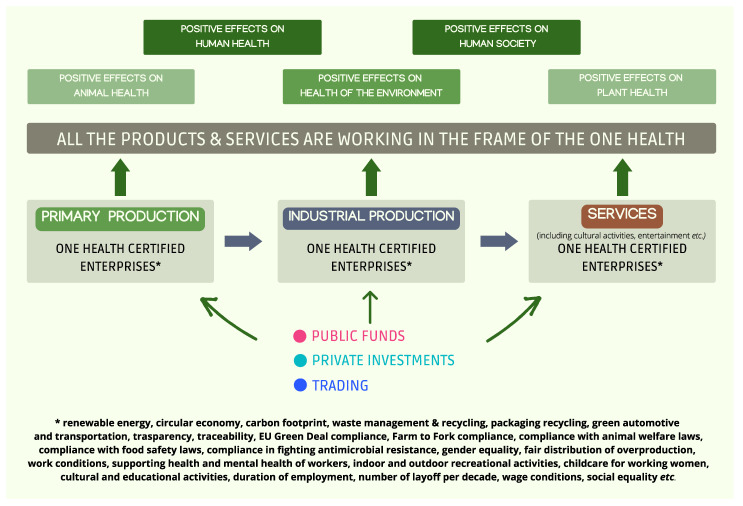
The in-embryo One Health Financial Model.

Recalling Dr. Bernard Url’s conclusive speech in the ONE2022 conference, “We have to change the financial model in which we are locked” [69]. Since investors bet on innovation only considering its impact/success [70] and the extent of economic return, we need to build a way to direct investments toward ethical and science-based choices. National and international rules concerning public/private investments and trading could regulate the flow of money, directing it exclusively toward companies that meet a broad range of OH parameters, ranging from environmental impact up to food safety, human health, work conditions, duration of employment, number of layoffs per decade, wage conditions, gender and social equality, etc. A OH conformity certificate for enterprises—listed in the stock market or not—should be awarded considering various aspects of the OH, each of which should be analysed using a proper checklist. Each analysed checklist could then be merged in a final document declaring (or not) the compliance of a specific enterprise with a minimum number of the considered parameters useful to evaluate its OH status. This in-embryo One Health Financial Model is based on money exclusively invested in certified enterprises. In this model, money can be effectively used in the effort to improve human society and global conditions, avoiding economic losses due to negative side effects on the holistically considered health, in the effort to effectively pursue the OH goal (Figure 2). Dedicated agencies and databanks preserving all data concerning certified enterprises are needed. Data—when shared in open access—could efficaciously improve public engagement, addressing consumers’ choices toward certified producers who comply with the OH objectives.

Despite the considerable efforts being carried out in the context of OH, war causes tragic and devastating effects on the physical and mental health of human beings; on their lives; on epidemic [71], pandemic, and zoonotic threats; on animals; on plants; and, last but not least, on the environment (Figure 1). War is incompatible with One Health. Enormous efforts for peace are therefore urgently needed.

## Data Availability

Not applicable.
